# Biomechanics Parameters of Gait Analysis to Characterize Parkinson’s Disease: A Scoping Review

**DOI:** 10.3390/s25020338

**Published:** 2025-01-09

**Authors:** Michela Russo, Marianna Amboni, Noemi Pisani, Antonio Volzone, Danilo Calderone, Paolo Barone, Francesco Amato, Carlo Ricciardi, Maria Romano

**Affiliations:** 1 Department of Electrical Engineering and Information Technology, University of Naples Federico II, 80125 Naples, Italy; michela.russo2@unina.it (M.R.); danilo.calderone@unina.it (D.C.); framato@unina.it (F.A.); mariarom@unina.it (M.R.); 2 Center for Neurodegenerative Diseases (CEMAND), Department of Medicine, Surgery and Dentistry “Scuola Medica Salernitana”, University of Salerno, 84131 Salerno, Italy; mamboni@unisa.it (M.A.); avolzone@unisa.it (A.V.); pbarone@unisa.it (P.B.); 3 Department of Advanced Biomedical Sciences, University of Naples Federico II, 80131 Naples, Italy; noemi.pisani@unina.it

**Keywords:** gait analysis, biomechanics, kinematics, kinetics, Parkinson’s disease

## Abstract

Parkinson’s disease (PD) is characterized by a slow, short-stepping, shuffling gait pattern caused by a combination of motor control limitations due to a reduction in dopaminergic neurons. Gait disorders are indicators of global health, cognitive status, and risk of falls and increase with disease progression. Therefore, the use of quantitative information on the gait mechanisms of PD patients is a promising approach, particularly for monitoring gait disorders and potentially informing therapeutic interventions, though it is not yet a well-established tool for early diagnosis or direct assessment of disease progression. Over the years, many studies have investigated the spatiotemporal parameters that are altered in the PD gait pattern, while kinematic and kinetic gait parameters are more limited. A scoping review was performed according to the PRISMA guidelines. The Scopus and PubMed databases were searched between 1999 and 2023. A total of 29 articles were included that reported gait changes in PD patients under different gait conditions: single free walking, sequential motor task, and dual task. The main findings of our review highlighted the use of optoelectronic systems for recording kinematic parameters and force plates for measuring kinetic parameters, due to their high accuracy. Most gait analyses in PD patients have been conducted at self-selected walking speeds to capture natural movement, although studies have also examined gait under various conditions. The results of our review indicated that PD patients experience alterations in the range of motion of the hip, knee, and ankle joints, as well as a reduction in the power generated/absorbed and the extensor/flexor moments. These findings suggest that the PD gait pattern may be more effectively understood using kinematic and kinetic parameters.

## 1. Introduction

Gait analysis is a field of research aimed at investigating the gait pattern and understanding the relationship between the functional capabilities and limitations of an individual, with the purpose of evaluating biomechanical performance and potentially preventing injuries in patients [1]. Gait analysis allows the objective measurement of gait cycle by using various quantitative features that are typically categorized into spatiotemporal, kinematic, and kinetic parameters [1,2,3].

Gait analysis is traditionally conducted in controlled environments using optical motion capture systems and force plates, which synchronize kinematic and kinetic data for detailed assessments [4]. However, newer approaches leverage low-cost infrared depth sensors and color cameras, like the Kinect, for markerless 3D analysis. Virtual reality also offers innovative possibilities for detailed movement evaluation [5]. Alternatively, wearable sensors (e.g., accelerometers and gyroscopes) and insole pressure sensors enable gait analysis outside the lab, promising practical applications in daily life settings [6].

Over the years, gait analysis has provided an essential way for the quantitative assessment of gait patterns, which are then used for the early diagnosis of disease, distinguishing between disease entities, monitoring of disease progression, and determining the severity, but also for predicting the outcome of interventions such as pharmacological therapies or rehabilitation [7,8,9,10]. In clinical and healthcare applications, gait analysis is a tool more and more adopted by physiotherapists, orthopedists, and neurologists [11,12].

Chen et al. [13] affirmed that, despite the wide spectrum of gait pathologies in medicine, studying the gait of parkinsonism has received the most research attention compared to other neurological conditions. This is likely due to the high prevalence of gait impairments in Parkinson’s disease (PD) and their significant impact on patients’ mobility and independence. Indeed, gait disorders are commonly observed in patients suffering from PD, and are often among the earliest and most disabling motor symptoms [14]. Clinical gait hallmarks of PD are slowness (bradykinesia), inability to perform a clinically perceivable movement (akinesia), or freezing of gait (FOG) [15]. These symptoms can lead to common gait limitations, such as irregular gait patterns, unbalance, and dual-task deficits [16,17,18]. Abnormalities in the spatiotemporal parameters of PD gait pattern have been widely discussed in the literature. For instance, Creaby et al. [19] reviewed the spatiotemporal characteristics of walking associated with an increased risk of future falls in individuals with PD. Similarly, Bouca-Machado et al. [20] conducted a systematic review to identify the most influential spatiotemporal parameters affecting PD gait.

Common abnormalities in the spatiotemporal parameters of gait in PD include reduced step length, decreased gait speed, and increased variability [11,21]. PD patients typically walk slowly, with reduced stride and cycle length, as well as an increased duration of double-limb support. These alterations contribute to postural instability and a higher risk of balance loss [22,23]. Such findings have been instrumental in developing diagnostic tools, monitoring disease progression, and evaluating the efficacy of therapeutic interventions [7,24,25].

To date, although a large number of studies have investigated the spatiotemporal parameters altered in the PD gait pattern [19,20,26,27,28], the attention to kinematic and kinetic gait variables of this disease is limited. In fact, each individual gait represents the final result of the combination of spatiotemporal, kinematic, and kinetic variables. Hence, depicting disease-specific gait patterns, specifically in PD, should not overlook kinematic and kinetic gait assessment.

This scoping review aimed to investigate how kinematic and kinetic parameters have been analyzed in PD patients during gait. In particular, we focused our interest on the experimental procedures, the experimental set-up, the main characteristics of the populations studied, and the main gait kinetic and kinematic parameters investigated by researchers. In addition, we attempted to summarize the main findings on kinematic and kinetic variables to characterize the PD-associated gait pattern.

## 2. Materials and Methods

This scoping review was conducted according to the PRISMA guidelines [29].

### 2.1. Search Strategy

To explore kinematic and kinetic gait changes in PD, we carried out a literature search in the PubMed and Scopus databases. For this study, publications selected from the research strategy starting in 1999 up to 2023 were considered, with the last database check conducted in June 2024.

The search key words were as follows: “Kinematics AND Kinetics AND Parkinson’s Disease”. All articles associated with idiopathic PD that investigated the biomechanical characteristics of gait focused on normal gait and that specified which kinematic and kinetic gait parameters had been studied were considered for inclusion.

### 2.2. Eligibility Criteria

The inclusion criteria for the study were as follows: studies had to assess straight-line walking and evaluate locomotion in either controlled or non-controlled environments. Additionally, they needed to measure biomechanical variables, specifically kinematic and/or kinetic data, and include participants who were men and/or women diagnosed with idiopathic PD.

On the other hand, the exclusion criteria ruled out case studies, review articles, books, book chapters, conference abstracts, editorials, and studies written in languages other than English. Also excluded were articles that investigated parkinsonisms other than idiopathic PD, such as atypical parkinsonism, or studies based on only healthy subjects, as well as those that did not involve a movement analysis of the lower limbs or did not specifically assess gait analysis. Furthermore, studies that only involved brief or single-step assessments were excluded. Continuous gait analysis is crucial for capturing the dynamic and persistent nature of gait disturbances in PD.

### 2.3. Study Selection

The researchers compiled a summary table to consolidate data from the selected articles. The final extraction included key information such as the title, journal, publication year, sample characteristics (including sample size and population type), study objectives, instrumentation, gait variables, and study outcomes. To minimize bias and enhance objectivity, two pairs of reviewers (M.R. (Michela Russo) and N.P.; D.C. and A.V.) independently conducted the initial screening without consulting others. In cases where the couple of the two reviewers disagreed on a record’s eligibility, a third pair of reviewers (C.R. and M.A.) was consulted to resolve the discrepancy by examining the contested records and making the final decision on study inclusion.

## 3. Results

**Figure 1:**
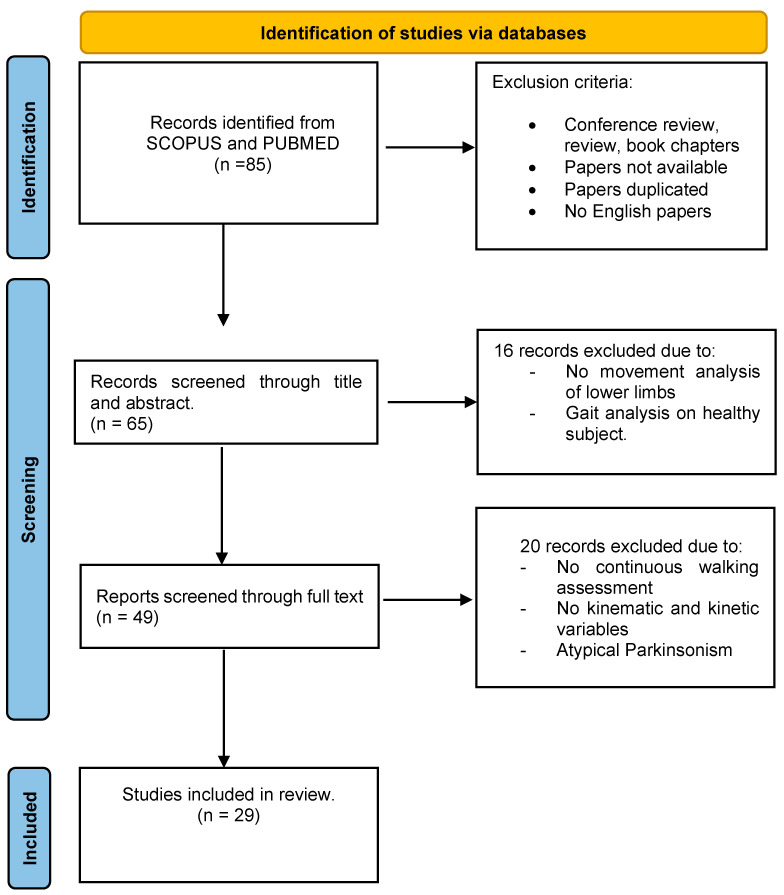
Description of the study selection process.

A total of 85 potential articles resulted from the PubMed and Scopus databases (62 in Scopus and 23 in PubMed). After removing duplicates, a total of 65 articles were screened against the inclusion/exclusion criteria. After reading the titles and abstracts, the full texts of 49 were assessed for eligibility. Overall, 29 articles were included in our study. Figure 1 shows a schematic representation of the selection process according to the PRISMA guidelines. Specific exclusion criteria were applied during the selection process: studies were excluded if they did not include gait analysis [30,31,32,33], did not focus on the lower limbs [31,32], or examined forms of parkinsonism other than idiopathic PD, such as atypical parkinsonism [34,35].

### Characteristics of the Included Studies

**Figure 2:**
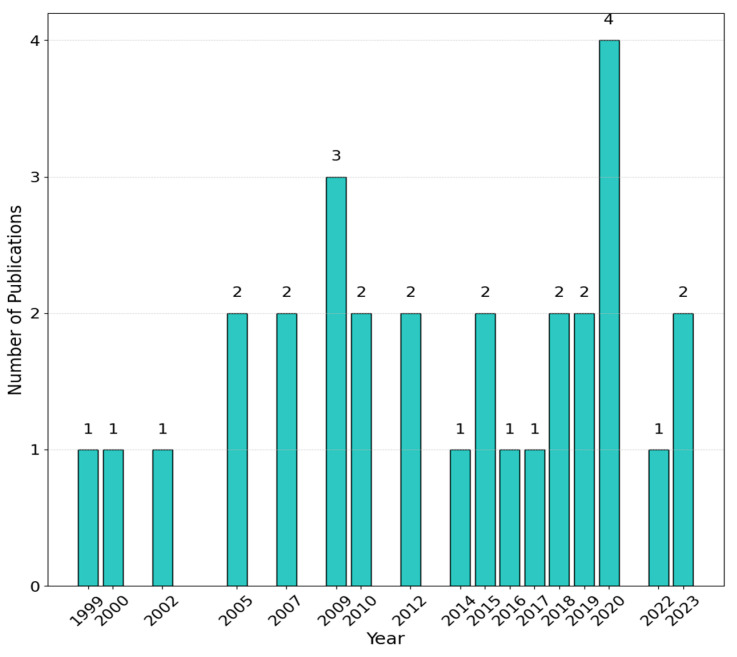
Annual distribution of the number of papers identified through the Scopus and PubMed databases.

In Figure 2, a histogram of the distribution of the papers that were published between 1999 and 2023 is shown. It is worth noting that the scientific studies meeting the inclusion criteria of our review were consistent over time. While the assessment of kinematic and kinetic gait changes using instrumented 3D gait analysis remains popular in the scientific literature due to its precision and comprehensive insights, the small number of studies may reflect barriers such as high costs, technical complexity, and the limited accessibility of 3D gait laboratories.

**Figure 3:**
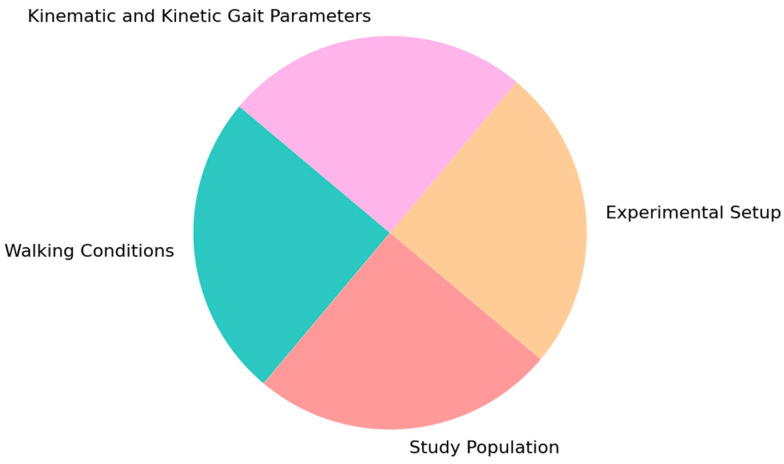
Categorization of studies included in this review: the studies were described based on key aspects such as the walking conditions, experimental set-up, study population, and kinematic and kinetic gait parameters.

According to the records found in the Scopus and PubMed databases, the authors focused on evaluating human locomotion using different instruments and in various gait conditions, providing accurate and precise quantitative measurements of the gait patterns and characteristics of PD patients. To enhance clarity, all papers were described using these categories, as shown in Figure 3: the walking conditions under investigation, the type of experimental set-up, the study population included, and the kinematic and kinetic parameters. These categories also correspond to the organization of the paragraphs presented in this review. Moreover, in the walking condition categories, the studies were also distinguished based on their primary objectives, such as assessing an external intervention like deep brain stimulation (DBS), or purely observing gait patterns, exploring the effects of visual and auditory cues, or examining gait in the context of cognitive interference, with a focus on understanding the relationship between mobility and cognitive functions.

## 4. Discussion

We conducted this scoping review to provide the relevant information available in the literature about the methodological aspects of gait analysis for investigating kinematic and kinetic gait alterations in PD patients.

### 4.1. Experimental Procedure/Walking Conditions

**Figure 4:**
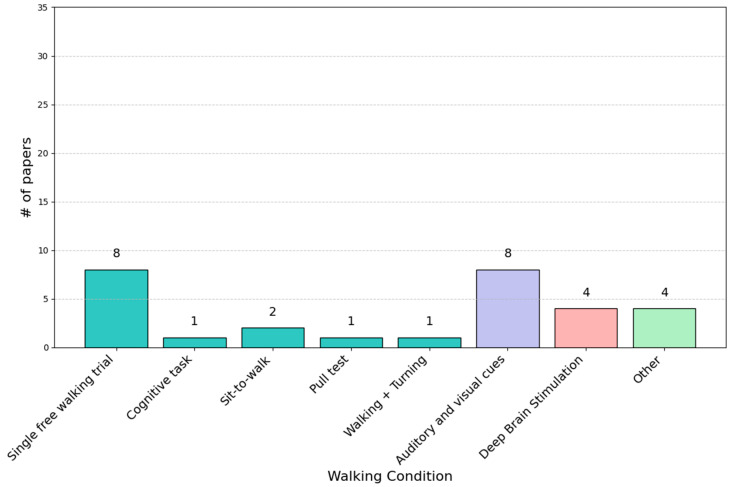
Percentage of articles for each walking condition. The figure categorizes study aspects based on single- and dual-task conditions, visual and auditory cues, and deep brain stimulation (DBS). The distribution of studies is represented using different colors: blue for single- and dual-task conditions, purple for visual and auditory cues, and pink for DBS. The green color indicates studies classified under the “Other” category.

Our research focused on examining the biomechanical changes in PD patients under various gait conditions. Figure 4 displays the proportion of articles dedicated to each type of walking condition. In this section, the main walking conditions are organized into three subsections:

1. Single- and Dual-Task Conditions: this category includes studies (*n* = 13) where PD gait patterns were assessed during free walking, sequential tasks, and dual tasks.
2. Visual and Auditory Cues: this category examines studies (*n* = 8) that investigate the impact of visual and auditory cues on the gait patterns of PD patients.
3. DBS-Related Walking Changes: this category focuses on studies (*n* = 4) that examine the gait characteristics of PD patients who have undergone DBS surgery.

An additional category, labeled “Other”, encompasses less common or less frequently studied (*n* = 4) conditions in gait analysis.

#### 4.1.1. Single Free Walking, Sequential Motor Task, and Dual-Task Conditions

PD patients may exhibit gait deficits during daily walking or when trying to perform a rapid sequential movement, such as moving from sitting to standing, crossing an obstacle, or performing two activities simultaneously. Therefore, it is crucial for clinicians to evaluate how PD patients walk during both routine and difficult activities in order to avoid falls and dangerous situations that could reduce their quality of life.

According to our findings, single free walking at a self-selected speed was the preferred task by researchers. Indeed, 27% of the articles explored the kinematics and kinetics of PD patients during this event [36,37,38,39,40,41,42,43].

Kleiner et al. [42] investigated how PD patients could interact with a supporting surface without slipping. They observed the influence of kinematic and kinetic gait variables on the coefficient of friction at the floor, which is calculated as the ratio of tangential to vertical GRF during the stance phase.

Indeed, it is known that PD patients are affected by hesitation during transitions in motor behavior and deficits in motor planning, commonly associated with executive dysfunction, when performing a sequential single task. For these reasons, several authors studied the response to a motor stimulus, such as sit-to-walk and sit-to-stand, the response to a push and pull, and turning while walking. Pelicioni et al. [44,45] studied motor function during a sit-to-walk task under time constraint, first in PD patients, as a whole, and then in different gait subtypes of PD. Likewise, McVey et al. [46] identified biomechanical variables that are sensitive to the effects of early PD on the ability to respond to a backwards waist pull. Similarly, Carpinella et al. [47] investigated the response in subjects with mild PD according to multitasking, including analysis of linear walking, gait initiation, and turning while walking. Participants were asked to walk at a self-selected speed for 2 m, turned to the left at an angle of 90°, and then continue walking in the new direction for another 2 m. Stegemöller et al. [48] exanimated differences in gait behavior and stability as PD patients walked across an obstacle along the walkway.

Linder et al. [5] examined the effects of an 8-week aerobic cycling intervention on the kinematics and kinetics of continuous walking, comparing the baseline and end of treatment. The authors hypothesized that a 50 min session of aerobic cycling for three times/week and for 8 weeks would induce improvements in gait biomechanics in a person with PD.

In addition, some studies investigated anticipatory postural adjustments (APAs). They precede movement transitions and are essential as they allow the unloading of the stepping leg, thus creating the conditions required for progression. Huffmaster et al. [49] examined the relationship between APAs preceding gait initiation and the kinematics of the first two steps in PD patients. Tard et al. [50] investigated how an attentional task carried out during step preparation can modulate APAs in PD patients with FOG (PD + FOG). Additionally, Bayot et al. [51] investigated at a behavioral and cortical level whether an attentional load can specifically impact the preparation and execution phases of step initiation in PD + FOG.

Furthermore, cognitive decline is common in PD, even in the early stages, and it occurs as dysfunction in executive, attention, memory, language, and visuospatial domains [52]. Given the close relationship between walking and cognitive processes [53], gait impairment in PD may be worse when combining walking with another task requiring cognitive engagement [54,55]. For these reasons, measuring the change in performance from a single to dual task could be indicative of cognitive reserve. The consequences of dual-tasks on gait have been largely studied using a wide range of concurrent cognitive tasks (e.g., generating words and counting backwards) [56]. For example, Nardello et al. [57] investigated the effects of a cognitive task on walking. Participants were instructed to walk the length of the walkway in two different conditions: normal walking and walking while performing a cognitive task (repetition of the weekdays in a reverse order).

#### 4.1.2. Visual and Auditory Cueing

The use of auditory and visual cues can improve gait in PD patients by compensating, with an external sensory aid (i.e., extrinsic driver), the loss of a self-generating motor plan (i.e., intrinsic driver) that is characteristically dysfunctional in PD patients and only partially counteracted by the frontal voluntary system [58,59]. Indeed, various researchers demonstrated that the use of visual cueing improves gait performances in spatiotemporal parameters as well as kinematic and kinetic performance at the ankle and hip joints.

In this review, 27% of articles investigated the influence of external temporal or spatial stimuli, namely auditory and visual cues [48,50,60,61,62,63,64,65]. In particular, Picelli et al. [62] investigated the effect of rhythmic auditory cues generated by a metronome on spatiotemporal, kinematic, and kinetic gait variables in PD patients under different conditions (uncued normal walking and 90%, 100%, and 110% of the mean cadence at preferred pace cued walking). Each patient was asked to maintain a uniform gait cadence to the auditory cue’s rhythm. Young et al. analyzed walking while PD patients underwent different auditory cues, such as verbal instruction, metronome, stepping, and footstep sounds [65]. As an alternative to auditory cues, several articles used visual cues. In particular, Morris et al. [61] investigated gait of PD patients in the presence and absence of a visual cue, which consisted of white strips of cardboard placed over a 10 m walkway at a distance representative of step length for a healthy control subject. Likewise, Lewis et al. [60] analyzed the kinematic and kinetic gait variables during two visual cue conditions. First, they used strips of white tape (5 × 50 cm) along the runway, perpendicular to the walking path. Then, they used a laser device that was attached to the subject’s chest that projected two laser lines on to the floor in front of the subject that were approximately 50 cm wide. In the course of time, the external stimuli allowed them to control and reduce one of the most severe motor symptoms, like FOG. Tang et al. [63] examined the changes in anatomic joints and the generated forces during gait performance in FOG PD patients under a laser cue condition. The laser cue condition was provided by a laser line projected from a laser generator attached over the sternum. Thus, patients were instructed to step on the laser line. Similarly, Egerton et al. [64] explored the biomechanical effect of a laser light visual cue on the center of pressure (COP) and center of mass (COM) during gait initiation in patients with PD and FOG. The authors demonstrated that the patient was able to synchronize movements with the visual cue, rather than being distracted by it.

#### 4.1.3. Deep Brain Stimulation

Several trials have tested the effectiveness of DBS as an intervention to improve motor symptoms in PD patients [66]. Electrical stimulation of the subthalamic nucleus (STN) has been shown to significantly improve motor symptoms and reduce dopaminergic therapy, leading to remarkable improvements in gait impairment [49]. Many authors suggested that the improvement in postural, gait, and balance control in PD patients could be ensured by DBS [67,68,69]. Indeed, the patient is able to provide a better response to destabilization with increased duration of agonist muscle activity. To date, many articles have conducted full quantitative gait assessments, showing that L-dopa alone was slightly less effective than STN-DBS, while the combination of STN stimulation and L-dopa further improved not only spatiotemporal parameters but also leg joint movements [70,71,72].

The research group of Rizzone and Ferrarin investigated the changes in gait parameters of PD patients during unilateral and bilateral DBS [73]. First, Ferrarin et al. [70] analyzed PD patients’ gait with bilaterally implanted STN stimulation devices. Walking performance was analyzed on the same day in four conditions: Stim Off–Med Off, Stim On–Med Off, Stim Off–Med On, and Stim On–Med On. Subsequently, Rizzone et al. [74] investigated the unilateral stimulation of a “dominant” STN, in order to demonstrate that it was capable of improving gait abnormalities, similarly to what can be observed in bilateral stimulation. Moreover, Johnsen et al. [75] showed that the effect of bilateral DBS of the STN improves PD gait and gait asymmetry.

### 4.2. Experimental Set-Up

**Figure 5:**
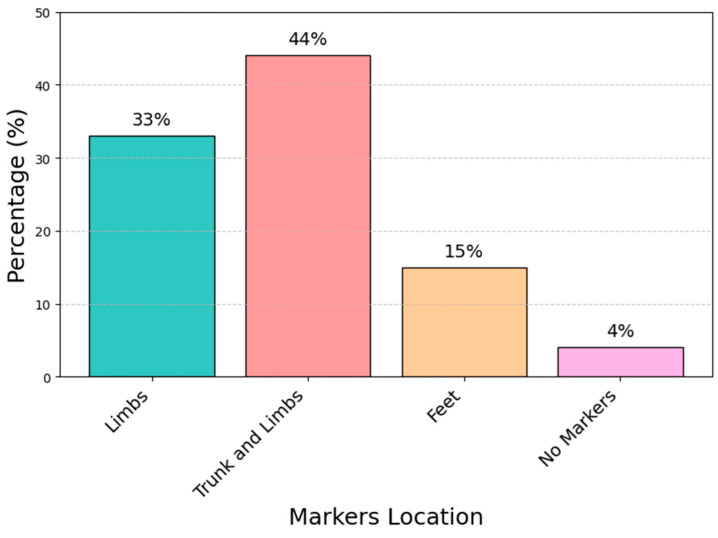
Percentages of articles for each marker’s placement on body area.

All the researchers conducted the gait analysis of PD patients in a controlled motion laboratory. In the majority of the studies (92%), kinematic variables were acquired using an infrared camera motion capture system with a minimum of two to a maximum of nine cameras, and kinetic variables were collected from force plates located halfway down the walkway, whose length ranged between 6 m and 20 m, with a minimum of a single to a maximum of four force plates. Infrared-reflexive markers were located on the anatomical bony landmarks, providing anatomical coordinates for the shoulders, arms, trunk, pelvis, hips, femur, tibia, and feet. The average number of spherical markers used in these studies ranged between a minimum of 8 and a maximum of 49. Figure 5 shows the percentage of articles for each marker’s placement on the body area. Both upper and lower anatomic areas were used in total in 44% of the studies. Other common positions are the position on both the lower limbs and both feet, found in 33% and 15% of the articles, respectively. The final category named “no marker” includes articles that either did not use markers or did not specify their use. Specifically, [41,42,62] used the Davis protocol [76], either in its full version with 22 markers or in a reduced version with 16 markers; [48,57] employed the Vicon Plug-in Gait model with a configuration of 39 markers, while other [36,39,49,63] did not specify the protocol or model used.

Our findings indicate that the location of markers in different body areas and the varying number of markers can introduce variability in marker placement. This discrepancy can lead to inconsistent data capture, especially if the markers are not placed accurately or uniformly across subjects. Variations in placement may affect the measurement of joint angles, gait parameters, and ultimately influence the interpretation of gait performance. Future research should consider the use of standardized marker sets to improve the comparability and reliability of results.

In contrast to the other studies, Eltouhky et al. and Oh et al. [38,40] showed that the Kinect system could be a feasible alternative to 3D motion analysis instruments in producing kinematic gait parameters. Additionally, Linder et al. [5] collected gait data using a three-dimensional motion capture system with an advanced virtual reality system widely used in the field of rehabilitation. Finally, Martinez et al. [37] quantified kinetic parameters using in-shoe pressure-sensitive insoles with 954 sensors. Detailed information on the experimental set-up and study population is shown in Table 1. The papers are cited in the table in the same way they are cited in the text.

### 4.3. Study Population/Demographic and Clinical Characteristics of Patients

**Figure 6:**
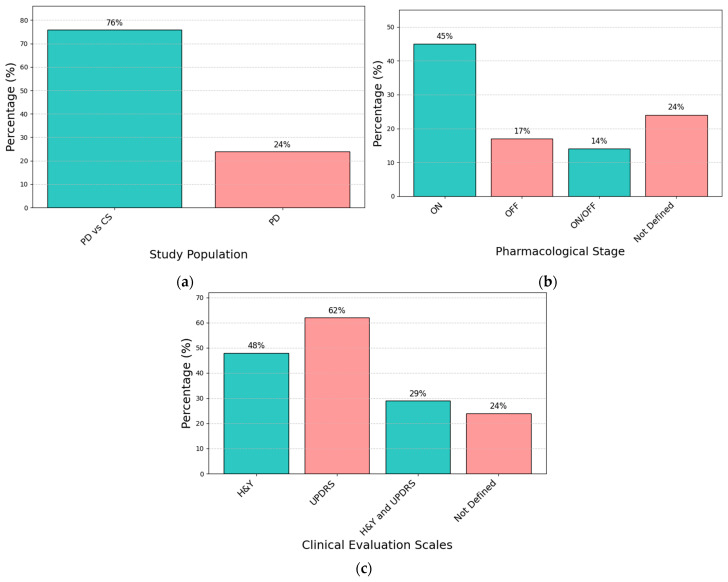
Percentage of articles for (**a**) study population: percentages of publications that investigate Parkinson’s disease (PD) patients and PD patients vs. control subjects (CS), (**b**) medication phase: percentage of publications that investigate PD patients during the on, off, and on/off medication phases, (**c**) evaluation scale: percentage of publications that investigate the Hoeh and Yahr (H&Y) scale, Unified Parkinsons’s Disease Rating Scale (UPDRS), and both evaluation scales.

Table 1 includes information on participant characteristics, such as the cohort for each study, presence of specific symptoms, pharmacological state, age, disease duration, and the evaluation scales (Figure 6 shows the percentages of articles for cohort, medication stage, and evaluation scales). The majority of the articles concerned idiopathic PD with an average age of 61.0 ± 6.9 years, 78% of them were related to comparing PD patients with matched healthy subjects [5,36,37,39,40,42,44,46,47,48,49,50,57,60,63,64,65,70,73,74], and 22% of the studies were related to comparing the PD population internally [38,45,61,62,75,77]. On average, studies included 14 PD patients, with a minimum of 1 and a maximum of 34. Most studies (48%) reported Hoehn and Yahr (H&Y) to evaluate the motor staging of disease [36,39,42,45,46,47,48,57,60,63,65,73,74]; additionally, or alternatively, a good number of studies (60%) used the Unified Parkinson’s Disease Rating Scale (UPDRS) as a clinical rating scale evaluating the severity of motor and non-motor symptoms in PD, while 33% of the articles reported both H&Y and UPDRS [5,36,39,42,45,46,47,57,63,65]. In the vast majority of articles (45%), gait was analyzed during the on phase of medication, namely 1–2 h after a medication dose, whereas only a small proportion of patients (17%) were tested during the off phase [5,39,42,63,64]. Shida et al. [43] investigated how dopaminergic therapy affects FOG in order to identify new therapeutic targets. The authors analyzed the interaction of FOG on the lower limb joint kinematics and ground reaction force parameters of gait in people with PD during both on and off dopaminergic dosing. Švehlík et al. [39] investigated quantitative gait analysis parameters in PD patients during off dopaminergic therapy in comparison with a group of healthy elderly controls, in order to better characterize kinematic and kinetic gait parameters in PD, independent of medication effect that might have confounding influence on gait patterns. Concerning gait disturbances, FOG is the most investigated symptom (about 20%) [42,43,49,50,63,64], followed by dystonia and hypokinesia [61].

### 4.4. Kinematic Parameters

**Figure 7:**
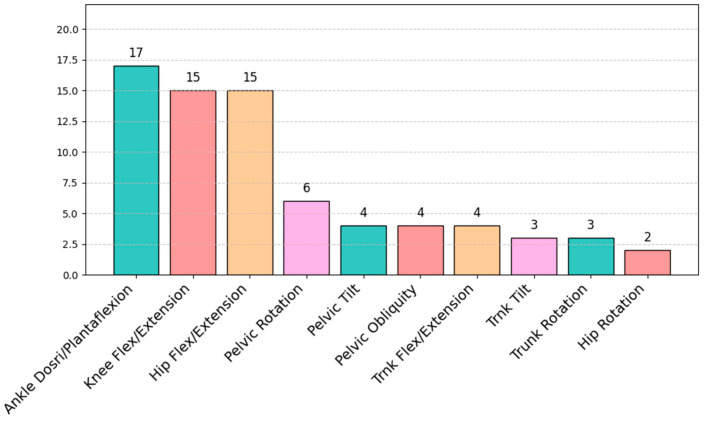
Distribution of kinematic parameters evaluated in the selected articles.

**Figure 8:**
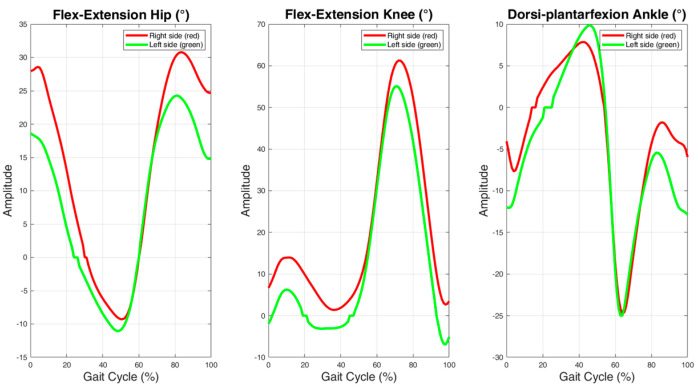
Kinematic parameters for the hip, knee, and ankle based on the sagittal plane. In red, the kinematic signal of the right side; in green, the kinematic signal of the left side.

Kinematic parameters based on the sagittal plane are the most reported variables in these studies, as shown in Figure 7. In particular, ankle dorsiflexion/plantarflexion, knee flexion/extension, and hip flexion/extension appear in the studies with a percentage of 58%, 50%, and 50%, respectively [5,36,39,43,46,61,62,63,73,74,75,77]. Joint movements in the sagittal plane are the most investigated and these are calculated by the authors in different phases of the gait cycle (e.g., initial contact, mid-stance phase, and swing phase); in Figure 8, we have reported the kinematic profiles for the hip, knee, and ankle joints and in Table 2 we have listed the most frequent joint or segment motions analyzed.

In addition, kinematic movements based on coronal and transverse planes, such as rotation and obliquity, have been investigated [36,39,41,43,47,70,73]. Carpinella et al. investigated the rotation and obliquity of the pelvic region, as well as forward inclination, lateral flexion, and rotation of trunk [47]. Likewise, Ferrarin et al. [70] analyzed the range of motion for each kinematic joint, such as pelvic tilt, obliquity, and rotation, and the range of motion for trunk torsion. Moreover, Castagna et al. [41] computed an index of jerkiness for all joints. Common kinematic findings are that PD gait is characterized by reduced angular movements at all lower-extremity joints. In particular, Sofuwa et al. [36] showed that the main changes were located at the ankle joint but not at the knee or the hip joints. Indeed, ankle plantarflexion at toe-off, range of motions (ROMs) at push-off, and swing phase were reduced in PD patients. Conversely, many researchers have shown that the ROMs are also reduced at the hip and the knee, beyond the ankle joints. Moreover, a number of studies show that PD patients walk with increased knee flexion during swing phase, reduced knee extension in the mid-stance phase, and reduced hip extension, as well as relevant reductions in the ROMs of the trunk and pelvic joints [36,39,41,47,70,73,74].

Therefore, these findings demonstrate that all major joints of the lower extremities of PD patients had a decreased ROM. In particular, PD patients showed a reduction at the knee, a loss of hip extension, and a decrease in ankle plantarflexion [36,39,61]. These alterations in the kinematic gait patterns were reflected in abnormal kinetics.

### 4.5. Kinetic Parameters

**Figure 9:**
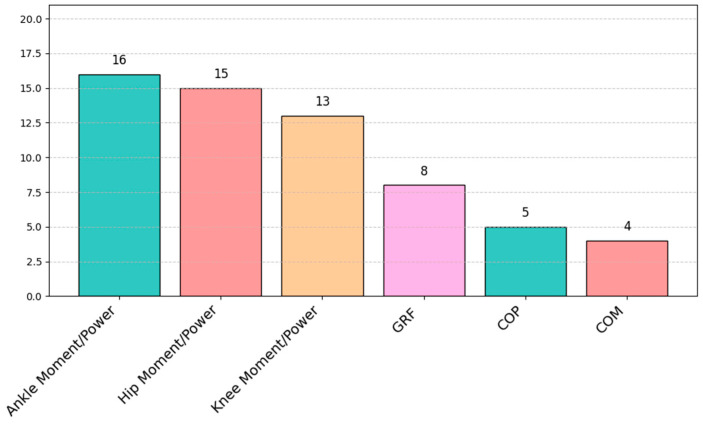
Distribution of kinetic parameters evaluated in the selected articles.

**Figure 10:**
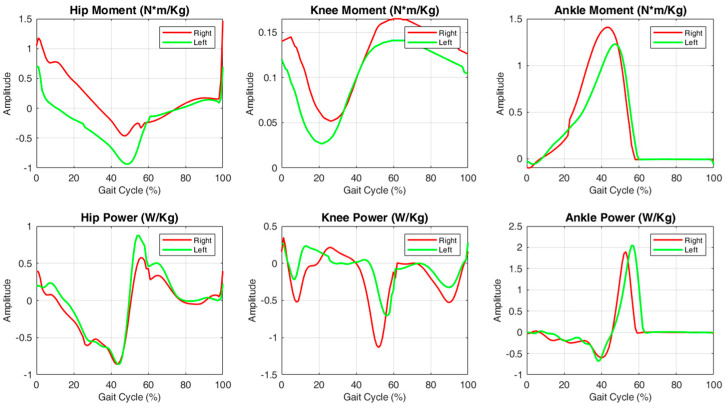
Kinetics parameters for the hip, knee, and ankle based on the sagittal plane. In red, the kinetic signal of the right side; in green, the kinetic signal of the left side.

Abnormal gait patterns in the PD patients were also evident in the kinetic parameters. The majority of articles investigated the moment and power of the ankle, hip, and knee joint movements in the sagittal plane (62%, 58%, and 50%, respectively), as shown in Figure 9. Additionally, some studies (20%) investigated balance control and its changes in PD patients [44,45,46,47,48,49,51,64]. Stegemöller et al. [48] investigated the interaction between COP and whole-body COM during obstacle crossing. McVey et al. determined the antero-posterior (A-P) and medio-lateral (M-L) COP displacements during the first step in the response to a backwards waist pull [46]. Similarly, Pelicioni et al. [44,45] investigated the A-P, M-L, and vertical COM range, COM velocity, and A-P, M-L and vertical moment during a sit-to-walk task. Furthermore, Evans et al. [30] analyzed the mean peak of the vertical, M-L, and A-P GRF during treadmill walking in PD patients. Martinez et al. [37] characterized the PD gait pattern, investigating the vertical force after the foot touches the ground, the final peak before toe-off, and the mid-stance force. In Figure 10, we have reported kinetic profiles for the hip, knee, and ankle joints; Table 3 shows the most frequent kinetic movements joint in different gait cycle phases (e.g., load response, mid and terminal stance, and pre-swing).

Overall, such research provided details on the kinetic pattern of PD patients. Švehlík et al. [22] showed a reduction in the ankle extension moment during stance phase and a reduced moment loading response, in agreement with the findings of Sofuwa et al. [19]. In addition, a reduced power generation was observed in the stance and pre-swing phases of PD gait. At the knee joint, PD patients showed a reduction in the extensor moment as well as in the flexor moment. Generally, PD patients exhibited less power generation in the single-support phase and less absorbed power in the stance phase. At the hip joint, the flexion moment, but not the extension moment, was reduced in PD patients. Moreover, PD patients showed a reduction in both generated and absorbed power [36,37,39,40]. Considering the GRF, Oh et al. [40] demonstrated that PD patients exhibited a lower peak in the vertical GRF and A-P GRF during propulsion, whereas Pelicioni et al. [44,45] affirmed that PD patients showed an increased M-L GRF to complete the sit-to-walk task. In addition, they showed that PD patients displayed a reduction in the A-P COM velocity and consequently a reduction in the A-P COM momentum, which may reflect motor planning deficits in PD patients.

## 5. Limitations

This review has certain limitations, including the potential for selection bias, as relevant studies in less common databases or published in other languages may have been inadvertently excluded. Additionally, the use of a limited set of keywords may have resulted in the omission of some relevant studies, and the heterogeneity in study designs, such as differences in population characteristics and measurement methods, limits the comparability and synthesis of findings across studies.

Although the number of articles is limited for each year, we have observed a continuous interest in this topic over the years. However, the presence of only one to five studies per year about 3D gait analysis in this population may be attributed to the high cost, technical complexity, and inaccessibility of 3D gait labs. Moreover, the exclusive use of laboratory-based equipment, without the presence of wearable sensors, may represent a limitation of this review, as it restricts assessment to controlled settings and may not fully capture gait characteristics in real-world environments.

Furthermore, the lack of a formal methodological quality assessment of the included studies, the absence of a meta-analysis, and the lack of prior protocol registration are additional limitations of this review. These factors may have restricted the depth of our analysis and the ability to draw more definitive conclusions.

## 6. Conclusions

This scoping review assessed the use of gait analysis to quantify kinematic and kinetic parameters in PD patients, focusing on experimental procedures, set-up, population characteristics, and key gait parameters. The main findings highlight the feasibility of using optoelectronic systems for recording kinematic parameters and force plates for measuring kinetic parameters due to their high accuracy. Most gait analyses in PD patients have been performed at self-selected walking speeds to capture natural movement, but it has also been examined under various conditions, including visual and/or auditory cues, obstacle walking, cognitive dual-task conditions, or using gait analysis to test external intervention, such as DBS.

The limited presence of wearables could be because optoelectronic labs, which are usually considered a gold standard for gait analysis, are more reliable for studying kinematics than wearables (tracing the movement of patients, maybe with markers, makes it easier and more reliable to study a flex-extension or an abd-adduction rather than using inertial sensors).

In conclusion, kinematic and kinetic findings help to understand pathological gait patterns in PD. However, it remains unclear which changes are due to the disease process and which may be compensatory, or treatment-related. Future reviews should consider whether the observed kinematic and kinetic changes are primarily a compensation for slowed gait speed or a direct consequence of the disease process, and which changes best reflect treatment effects. Longitudinal assessments in large PD cohorts from early stages could provide valuable insights into gait pathogenesis and enable earlier, more effective therapeutic interventions.

## Figures and Tables

**Table 1:** Characteristics of experimental set-up and study population.

Author/Year	Experimental Set-Up	Walkway	Markers Set-Up (Number of Markers)	Sample Size Cohort	Medication State	H&Y	UPDRS	Age
Linder et al., 2022 [5]	Virtual reality system	-	-	14 PD vs. 14 CS	Off medication dose	-	35.0 ± 10.4	64.9 ± 5.5
Sofuwa et al., 2005 [36]	8 cameras 3 force plates	8 m	Limbs	15 PD vs. 9 CS	On medication dose	2.6 ± 0.6	16.1 ± 6.4	63.1 ± 8.4
Martínez et al., 2018 [37]	Sensor pressure insoles	25 m	Feet	16 young CS vs. 11 PD vs. 20 age-matched CS	On medication dose	-	15 ± 4.3	57 ± 7.8
Eltoukhy et al., 2017 [38]	8 cameras Kinect sensor	-	Limbs (16)	9 PD	-	-	-	71.0 ± 5.6
Švehlík et al., 2009 [39]	2 cameras 4 force plates	12 m	Limbs (8)	20 PD vs. 20 CS	Off medication dose	2.5	37.9	50 to 80
Oh et al., 2020 [40]	Camera Kinect sensor	5 m	-	9 PD vs. 11 CS	-	-	-	71.0 ± 5.6
Castagna et al., 2016 [41]	8 cameras 4 force plates	8 m	Trunk and limbs (22)	15 PD vs. 15 CS	On/off medication dose	-	On = 15.2 ± 10.6 Off = 27.7 ± 12.5	50.7 ± 11.5
Kleiner et al., 2018 [42]	6 cameras 1 force plate	8 m	Trunk and limbs (22)	14 PD vs. 14 CS	Off medication dose	2.4 ± 0.5	23.8 ± 5.0	65.9 ± 8.5
Shida et al., 2023 [43]	12 cameras 5 force plates	10 m	Trunk and limbs (44)	11 PD + FOG vs. 11 PD − FOG vs. 18 CS	On/off medication dose	2.3 ± 0.7	PD − FOG = 24.8 ± 13.6 PD + FOG = 26.4 ± 11.8	-
Pelicioni et al., 2019 [44]	4 cameras 2 force plates	-	Limbs (22)	14 PD vs. 14 CS	On medication dose		27.2 ± 6.8	70.7 ± 4.5
Pelicioni et al., 2020 [45]	4 cameras 2 force plates	-	Trunk and limbs (22)	USS PD	On medication dose	1.1 ± 0.9	22.1 ± 0.4	-
McVey et al., 2009 [46]	6 cameras 3 force plates	-	Limbs	10 PD vs. 10 CS	On medication dose	2.0	27.3 ± 15.0	48.0 to 77.0
Carpinella et al., 2007 [47]	9 cameras 4 force plates	6 m	Trunk and limbs (29)	7 PD vs. 7 CS	On medication dose	1.5	5.6 ± 3.0	65.9 ± 4.8
Stegemöller et al. 2012 [48]	8 cameras 2 force plates	9 m	Trunk and limbs (39)	10 PD vs. 10 CS	On medication dose	2.4 ± 0.6	-	62.0 ± 9.3
Huffmaster et al. 2020 [49]	8 cameras 1 force plate	-	Feet	11 PD + FOG vs. 14 PD − FOG	-	-	-	-
Tard et al., 2014 [50]	6 cameras 2 force plates	-	Feet	15 PD + FOG vs. 15 PD − FOG vs. 15 CS	-	-	PD − FOG = 20.7 ± 9.4 PD + FOG = 18.6 ± 9.3	PD − FOG = 60.6 ± 9.5 PD + FOG= 62.2 ± 6.3
Bayot et al., 2023 [51]	1 force plate	10 m	-	15 PD + FOG vs. 16 PD − FOG vs. 15 CS	On medication dose	2.0 ± 1.0	PD + FOG = 29.0 ± 1.0 PD − FOG = 23.0 ± 1.0	PD + FOG = 65.0 ± 6.0 PD − FOG = 62.0 ± 9.0
Nardello et al., 2020 [57]	8 cameras 1 force plates	8 m	Trunk and limbs (49)	10 PD vs. 10 CS	-	1.1 ± 0.9	22.1 ± 0.4	66.9 ± 7.0
Lewis et al., 2000 [60]	5 cameras 2 force plates	-	Trunk and limbs (19)	14 PD vs. 14 CS	On medication dose	2.8 ± 0.8	-	71.3 ± 7.6
Morris et al., 1999 [61]	4 cameras 1 force plate	20 m	Limbs (13)	USS PD	On/off medication dose	-	-	71.0
Picelli et al., 2010 [62]	6 cameras 1 force plate	10 m	Limbs (16)	8 PD	On medication dose	-	21.8 ± 1.9	65.1 ± 4.7
Tang et al., 2019 [63]	8 cameras 3 force plates	10 m	Limbs	34 PD vs. 32 CS	Off medication dose	2.4 ± 0.8	25.8 ± 2.7	71.4 ± 8.1
Egerton et al., 2015 [64]	10 cameras 3 force plates	-	-	1 PD + FOG vs. 1 CS	Off medication dose	-	-	-
Young et al., 2014 [65]	-	12 m	Feet	10 PD vs. CS	On medication dose	2.2	29.9 ± 11.5	64.6 ± 5.0
Ferrarin et al., 2005 [70]	4 cameras 1 force plate	10 m	Trunk and limbs (19)	10 PD vs. 10 CS	-	-	s − m− = 61.6 ± 10.9 s + m− = 20.5 ± 10.1 s − m+ = 24.4 ± 14.4 s + m+ = 13.2 ± 7.3	52.0 to 68.0
Rizzone et al., 2002 [73]	4 cameras 1 force plate	10 m	Trunk and limbs (19)	9 PD vs. 9 CS	-	3.6 ± 0.7	-	-
Rizzone et al., 2017 [74]	4 cameras 1 force plate	10 m	Limbs	10 PD vs. 10 CS	12 h after medication dose	3.7 ± 0.7	-	60.2 ± 4.8
Johnsen et al., 2009 [75]	8 cameras 1 force plates	10 m	Trunk and limbs (39)	12 PD	On/off medication dose	-	-	-
Evans et al., 2007 [77]	Camera Force platform	-	Trunk and limbs	1 PD	On medication dose	-	-	54.0

Parkinson’s disease (PD); control subject (CS); freezing of gate (FOG); PD patient with FOG (PD + FOG); PD patient without FOG (PD − FOG); Stimulation and Medication (s+m+), Stimulation without Medication (s + m−), Medication without Stimulation (s-m+), without Medication without Stimulation (m − s−); information not available (−).; Hoehn and Yahr scale (H&Y); Unified Parkinson’s Disease Rating Scale (UPDRS); Unspecified Sample Size (USS). The papers are cited in the table in the same way they are cited in the text.

**Table 2:** Movements on sagittal plane for each joint in different gait cycle phases.

	Ankle	Knee	Hip
Flexion/Extension Initial contact	x	x	x
Dorsiflexion in stance	x		
Plantarflexion at toe-off	x		
Plantarflexion in swing	x		
Dorsiflexion in swing	x		
Flexion in stance		x	
Extension in stance		x	x
Flexion at toe-off		x	x
Flexion in swing		x	x
Jerk index in stance	x	x	x
Jerk index in swing	x	x	x

**Table 3:** Kinetic movements for each joint in different gait cycle phases.

	Ankle	Knee	Hip
Moment at loading response	x	x	x
Moment in mid and terminal stance	x		
Moment in pre-swing	x		
Power absorption at loading response	x		
Power generation at pre-swing	x		
Flexion moment in stance		x	x
Extension moment in stance		x	x
Abduction moment in stance			x
Power generated in stance		x	x
Power absorbed in late stance		x	x

## Data Availability

No new data were created.

## References

[1] Akhtaruzzaman M., Shafie A.A., Khan M.R. (2016). Gait Analysis: Systems, Technologies, and Importance. J. Mech. Med. Biol..

[2] Dicharry J. (2010). Kinematics and Kinetics of Gait: From Lab to Clinic. Clin. Sports Med..

[3] Webster J.B., Darter B.J., Webster J.B., Murphy D.P. (2019). 4—Principles of Normal and Pathologic Gait. Atlas of Orthoses and Assistive Devices (Fifth Edition).

[4] Cappozzo A., Della Croce U., Leardini A., Chiari L. (2005). Human Movement Analysis Using Stereophotogrammetry: Part 1: Theoretical Background. Gait Posture.

[5] Linder S.M., Baron E., Learman K., Koop M.M., Penko A., Espy D., Streicher M., Alberts J.L. (2022). An 8-Week Aerobic Cycling Intervention Elicits Improved Gait Velocity and Biomechanics in Persons with Parkinson’s Disease. Gait Posture.

[6] Prasanth H., Caban M., Keller U., Courtine G., Ijspeert A., Vallery H., von Zitzewitz J. (2021). Wearable Sensor-Based Real-Time Gait Detection: A Systematic Review. Sensors.

[7] Baker R. (2006). Gait Analysis Methods in Rehabilitation. J. NeuroEng. Rehabil..

[8] Russo M., Ricciardi C., Amboni M., Volzone A., Barone P., Romano M., Francesco A. A Cluster Analysis for Parkinson’s Disease Phenotyping with Gait Parameters. Proceedings of the 2023 IEEE International Conference on Metrology for eXtended Reality, Artificial Intelligence and Neural Engineering (MetroXRAINE).

[9] Russo M., Amboni M., Barone P., Pellecchia M.T., Romano M., Ricciardi C., Amato F. (2023). Identification of a Gait Pattern for Detecting Mild Cognitive Impairment in Parkinson’s Disease. Sensors.

[10] Russo M., Ricciardi C., Amboni M., Picillo M., Ricciardelli G., Abate F., Tepedino M.F., Calabrese M.C., Cesarelli M., Romano M. Performing a Short Sway to Distinguish Parkinsonisms. Proceedings of the 2022 IEEE International Conference on Metrology for Extended Reality, Artificial Intelligence and Neural Engineering (MetroXRAINE).

[11] Moon Y., Sung J., An R., Hernandez M.E., Sosnoff J.J. (2016). Gait Variability in People with Neurological Disorders: A Systematic Review and Meta-Analysis. Hum. Mov. Sci..

[12] Mayich D.J., Novak A., Vena D., Daniels T.R., Brodsky J.W. (2014). Gait Analysis in Orthopedic Foot and Ankle Surgery—Topical Review, Part 1: Principles and Uses of Gait Analysis. Foot Ankle Int..

[13] Chen S., Lach J., Lo B., Yang G.-Z. (2016). Toward Pervasive Gait Analysis With Wearable Sensors: A Systematic Review. IEEE J. Biomed. Health Inform..

[14] Chen P.-H., Wang R.-L., Liou D.-J., Shaw J.-S. (2013). Gait Disorders in Parkinson’s Disease: Assessment and Management. Int. J. Gerontol..

[15] Antony P.M.A., Diederich N.J., Krüger R., Balling R. (2013). The Hallmarks of Parkinson’s Disease. FEBS J..

[16] Baiano C., Barone P., Trojano L., Santangelo G. (2020). Prevalence and Clinical Aspects of Mild Cognitive Impairment in Parkinson’s Disease: A Meta-Analysis. Mov. Disord..

[17] Moore O., Peretz C., Giladi N. (2007). Freezing of Gait Affects Quality of Life of Peoples with Parkinson’s Disease beyond Its Relationships with Mobility and Gait. Mov. Disord. Off. J. Mov. Disord. Soc..

[18] Schrag A., Jahanshahi M., Quinn N. (2000). What Contributes to Quality of Life in Patients with Parkinson’s Disease?. J. Neurol. Neurosurg. Psychiatry.

[19] Creaby M.W., Cole M.H. (2018). Gait Characteristics and Falls in Parkinson’s Disease: A Systematic Review and Meta-Analysis. Park. Relat. Disord..

[20] Bouça-Machado R., Jalles C., Guerreiro D., Pona-Ferreira F., Branco D., Guerreiro T., Matias R., Ferreira J.J. (2020). Gait Kinematic Parameters in Parkinson’s Disease: A Systematic Review. J. Park. Dis..

[21] Amboni M., Iuppariello L., Iavarone A., Fasano A., Palladino R., Rucco R., Picillo M., Lista I., Varriale P., Vitale C. (2018). Step Length Predicts Executive Dysfunction in Parkinson’s Disease: A 3-Year Prospective Study. J. Neurol..

[22] Hausdorff J.M., Cudkowicz M.E., Firtion R., Wei J.Y., Goldberger A.L. (1998). Gait Variability and Basal Ganglia Disorders: Stride-to-Stride Variations of Gait Cycle Timing in Parkinson’s Disease and Huntington’s Disease. Mov. Disord..

[23] Morris M.E., Huxham F., McGinley J., Dodd K., Iansek R. (2001). The Biomechanics and Motor Control of Gait in Parkinson Disease. Clin. Biomech..

[24] Mikolajczyk T., Ciobanu I., Badea D.I., Iliescu A., Pizzamiglio S., Schauer T., Seel T., Seiciu P.L., Turner D.L., Berteanu M. (2018). Advanced Technology for Gait Rehabilitation: An Overview. Adv. Mech. Eng..

[25] di Biase L., Di Santo A., Caminiti M.L., De Liso A., Shah S.A., Ricci L., Di Lazzaro V. (2020). Gait Analysis in Parkinson’s Disease: An Overview of the Most Accurate Markers for Diagnosis and Symptoms Monitoring. Sensors.

[26] Amboni M., Ricciardi C., Picillo M., De Santis C., Ricciardelli G., Abate F., Tepedino M.F., D’Addio G., Cesarelli G., Volpe G. (2021). Gait Analysis May Distinguish Progressive Supranuclear Palsy and Parkinson Disease since the Earliest Stages. Sci. Rep..

[27] Amboni M., Ricciardi C., Cuoco S., Donisi L., Volzone A., Ricciardelli G., Pellecchia M.T., Santangelo G., Cesarelli M., Barone P. (2022). Mild Cognitive Impairment Subtypes Are Associated With Peculiar Gait Patterns in Parkinson’s Disease. Front. Aging Neurosci..

[28] Picillo M., Ricciardi C., Tepedino M.F., Abate F., Cuoco S., Carotenuto I., Erro R., Ricciardelli G., Russo M., Cesarelli M. (2021). Gait Analysis in Progressive Supranuclear Palsy Phenotypes. Front. Neurol..

[29] Page M.J., McKenzie J.E., Bossuyt P.M., Boutron I., Hoffmann T.C., Mulrow C.D., Shamseer L., Tetzlaff J.M., Akl E.A., Brennan S.E. (2021). The PRISMA 2020 Statement: An Updated Guideline for Reporting Systematic Reviews. BMJ.

[30] Mak M.K.Y., Hui-Chan C.W.Y. (2004). Audiovisual Cues Can Enhance Sit-to-Stand in Patients with Parkinson’s Disease. Mov. Disord..

[31] Rand M.K., Stelmach G.E., Bloedel J.R. (2000). Movement Accuracy Constraints in Parkinson’s Disease Patients. Neuropsychologia.

[32] Bronte-Stewart H.M., Ding L., Alexander C., Zhou Y., Moore G.P. (2000). Quantitative Digitography (QDG): A Sensitive Measure of Digital Motor Control in Idiopathic Parkinson’s Disease. Mov. Disord..

[33] Foreman K.B., Singer M.L., Addison O., Marcus R.L., LaStayo P.C., Dibble L.E. (2014). Effects of Dopamine Replacement Therapy on Lower Extremity Kinetics and Kinematics during a Rapid Force Production Task in Persons with Parkinson Disease. Gait Posture.

[34] Leiguarda R., Merello M., Balej J., Starkstein S., Nogues M., Marsden C.D. (2000). Disruption of Spatial Organization and Interjoint Coordination in Parkinson’s Disease, Progressive Supranuclear Palsy, and Multiple System Atrophy. Mov. Disord..

[35] Ali F., Loushin S.R., Botha H., Josephs K.A., Whitwell J.L., Kaufman K. (2021). Laboratory Based Assessment of Gait and Balance Impairment in Patients with Progressive Supranuclear Palsy. J. Neurol. Sci..

[36] Sofuwa O., Nieuwboer A., Desloovere K., Willems A.-M., Chavret F., Jonkers I. (2005). Quantitative Gait Analysis in Parkinson’s Disease: Comparison With a Healthy Control Group. Arch. Phys. Med. Rehabil..

[37] Martínez M., Villagra F., Castellote J.M., Pastor M.A. (2018). Kinematic and Kinetic Patterns Related to Free-Walking in Parkinson’s Disease. Sensors.

[38] Eltoukhy M., Kuenze C., Andersen M.S., Oh J., Signorile J. (2017). Prediction of Ground Reaction Forces for Parkinson’s Disease Patients Using a Kinect-Driven Musculoskeletal Gait Analysis Model. Med. Eng. Phys..

[39] Švehlík M., Zwick E.B., Steinwender G., Linhart W.E., Schwingenschuh P., Katschnig P., Ott E., Enzinger C. (2009). Gait Analysis in Patients With Parkinson’s Disease Off Dopaminergic Therapy. Arch. Phys. Med. Rehabil..

[40] Oh J., Eltoukhy M., Kuenze C., Andersen M.S., Signorile J.F. (2020). Comparison of Predicted Kinetic Variables between Parkinson’s Disease Patients and Healthy Age-Matched Control Using a Depth Sensor-Driven Full-Body Musculoskeletal Model. Gait Posture.

[41] Castagna A., Frittoli S., Ferrarin M., Del Sorbo F., Romito L.M., Elia A.E., Albanese A. (2016). Quantitative Gait Analysis in Parkin Disease: Possible Role of Dystonia. Mov. Disord..

[42] Kleiner A., Cubillos Arcila D., Pinto C., Salazar A.P., Marchese R., Barros R., Galli M., Pagnussat A. (2018). The Required Coefficient of Friction in Parkinson’s Disease: People with Freezing of Gait. Funct. Neurol..

[43] Shida T.K.F., de Oliveira C.E.N., da Silva Fragoso de Campos D., Los Angeles E., Bernardo C., dos Santos de Oliveira L., Salloum e Silva L.C., Novaes T.M., Shokur S., Bouri M. (2023). Effect of Freezing of Gait and Dopaminergic Medication in the Biomechanics of Lower Limbs in the Gait of Patients with Parkinson’s Disease Compared to Neurologically Healthy. Neurosci. Lett..

[44] Pelicioni P.H.S., Pereira M.P., Lahr J., Rodrigues M.M.L., de Morais L.C., Moraes R., Gobbi L.T.B. (2019). Motor Adjustments during Time-Constrained Sit-to-Walk in People with Parkinson’s Disease. Exp. Gerontol..

[45] Pelicioni P.H.S., Pereira M.P., Lahr J., Rodrigues M.M.L., Gobbi L.T.B. (2020). Biomechanical Analysis of Sit-to-Walk in Different Parkinson’s Disease Subtypes. Clin. Biomech..

[46] McVey M.A., Stylianou A.P., Luchies C.W., Lyons K.E., Pahwa R., Jernigan S., Mahnken J.D. (2009). Early Biomechanical Markers of Postural Instability in Parkinson’s Disease. Gait Posture.

[47] Carpinella I., Crenna P., Calabrese E., Rabuffetti M., Mazzoleni P., Nemni R., Ferrarin M. (2007). Locomotor Function in the Early Stage of Parkinson’s Disease. IEEE Trans. Neural Syst. Rehabil. Eng..

[48] Stegemöller E.L., Buckley T.A., Pitsikoulis C., Barthelemy E., Roemmich R., Hass C.J. (2012). Postural Instability and Gait Impairment During Obstacle Crossing in Parkinson’s Disease. Arch. Phys. Med. Rehabil..

[49] Amundsen Huffmaster S.L., Lu C., Tuite P.J., MacKinnon C.D. (2020). The Transition from Standing to Walking Is Affected in People with Parkinson’s Disease and Freezing of Gait. J. Park. Dis..

[50] Tard C., Dujardin K., Bourriez J.-L., Destée A., Derambure P., Defebvre L., Delval A. (2014). Attention Modulates Step Initiation Postural Adjustments in Parkinson Freezers. Park. Relat. Disord..

[51] Bayot M., Dujardin K., Gérard M., Braquet A., Tard C., Betrouni N., Defebvre L., Delval A. (2023). The Contribution of Executive Control Dysfunction to Freezing of Gait in Parkinson’s Disease. Clin. Neurophysiol..

[52] Barone P., Aarsland D., Burn D., Emre M., Kulisevsky J., Weintraub D. (2011). Cognitive Impairment in Nondemented Parkinson’s Disease. Mov. Disord. Off. J. Mov. Disord. Soc..

[53] Amboni M., Barone P., Hausdorff J.M. (2013). Cognitive Contributions to Gait and Falls: Evidence and Implications. Mov. Disord. Off. J. Mov. Disord. Soc..

[54] Kelly V.E., Eusterbrock A.J., Shumway-Cook A. (2011). A Review of Dual-Task Walking Deficits in People with Parkinson’s Disease: Motor and Cognitive Contributions, Mechanisms, and Clinical Implications. Park. Dis..

[55] Russo M., Amboni M., Volzone A., Cuoco S., Camicioli R., Di Filippo F., Barone P., Romano M., Amato F., Ricciardi C. (2024). Kinematic and Kinetic Gait Features Associated With Mild Cognitive Impairment in Parkinson’s Disease. IEEE Trans. Neural Syst. Rehabil. Eng..

[56] Raffegeau T.E., Krehbiel L.M., Kang N., Thijs F.J., Altmann L.J.P., Cauraugh J.H., Hass C.J. (2019). A Meta-Analysis: Parkinson’s Disease and Dual-Task Walking. Park. Relat. Disord..

[57] Nardello F., Bombieri F., Monte A. (2020). Leverage Mechanical Alterations during Walking at Self-Selected Speed in Patients with Parkinson’s Disease. Gait Posture.

[58] Suteerawattananon M., Morris G.S., Etnyre B.R., Jankovic J., Protas E.J. (2004). Effects of Visual and Auditory Cues on Gait in Individuals with Parkinson’s Disease. J. Neurol. Sci..

[59] Rochester L., Hetherington V., Jones D., Nieuwboer A., Willems A.-M., Kwakkel G., Van Wegen E. (2005). The Effect of External Rhythmic Cues (Auditory and Visual) on Walking During a Functional Task in Homes of People With Parkinson’s Disease. Arch. Phys. Med. Rehabil..

[60] Lewis G.N., Byblow W.D., Walt S.E. (2000). Stride Length Regulation in Parkinson’s Disease: The Use of Extrinsic, Visual Cues. Brain.

[61] Morris M.E., McGinley J., Huxham F., Collier J., Iansek R. (1999). Constraints on the Kinetic, Kinematic and Spatiotemporal Parameters of Gait in Parkinson’s Disease. Hum. Mov. Sci..

[62] Picelli A., Camin M., Tinazzi M., Vangelista A., Cosentino A., Fiaschi A., Smania N. (2010). Three-Dimensional Motion Analysis of the Effects of Auditory Cueing on Gait Pattern in Patients with Parkinson’s Disease: A Preliminary Investigation. Neurol. Sci..

[63] Tang L., Xu W., Li Z., Chen Y., Chen H., Yu R., Zhu X., Gu D. (2019). Quantitative Gait Analysis for Laser Cue in Parkinson’s Disease Patients with Freezing of Gait. Ann. Transl. Med..

[64] Egerton C.J., McCandless P., Evans B., Janssen J., Richards J.D. (2015). Laserlight Visual Cueing Device for Freezing of Gait in Parkinson’s Disease: A Case Study of the Biomechanics Involved. Physiother. Theory Pract..

[65] Young W.R., Rodger M.W.M., Craig C.M. (2014). Auditory Observation of Stepping Actions Can Cue Both Spatial and Temporal Components of Gait in Parkinson׳s Disease Patients. Neuropsychologia.

[66] Kurtis M.M., Rajah T., Delgado L.F., Dafsari H.S. (2017). The Effect of Deep Brain Stimulation on the Non-Motor Symptoms of Parkinson’s Disease: A Critical Review of the Current Evidence. npj Park. Dis..

[67] Cossu G., Pau M. (2017). Subthalamic Nucleus Stimulation and Gait in Parkinson’s Disease: A Not Always Fruitful Relationship. Gait Posture.

[68] Roper J.A., Kang N., Ben J., Cauraugh J.H., Okun M.S., Hass C.J. (2016). Deep Brain Stimulation Improves Gait Velocity in Parkinson’s Disease: A Systematic Review and Meta-Analysis. J. Neurol..

[69] Pötter-Nerger M., Volkmann J. (2013). Deep Brain Stimulation for Gait and Postural Symptoms in Parkinson’s Disease. Mov. Disord..

[70] Ferrarin M., Rizzone M., Bergamasco B., Lanotte M., Recalcati M., Pedotti A., Lopiano L. (2005). Effects of Bilateral Subthalamic Stimulation on Gait Kinematics and Kinetics in Parkinson’s Disease. Exp. Brain Res..

[71] Lubik S., Fogel W., Tronnier V., Krause M., König J., Jost W.H. (2006). Gait Analysis in Patients with Advanced Parkinson Disease: Different or Additive Effects on Gait Induced by Levodopa and Chronic STN Stimulation. J. Neural Transm..

[72] Mera T., Vitek J.L., Alberts J.L., Giuffrida J.P. (2011). Kinematic Optimization of Deep Brain Stimulation across Multiple Motor Symptoms in Parkinson’s Disease. J. Neurosci. Methods.

[73] Rizzone M., Ferrarin M., Pedotti A., Bergamasco B., Bosticco E., Lanotte M., Perozzo P., Tavella A., Torre E., Recalcati M. (2002). High-Frequency Electrical Stimulation of the Subthalamic Nucleus in Parkinson’s Disease: Kinetic and Kinematic Gait Analysis. Neurol. Sci..

[74] Rizzone M.G., Ferrarin M., Lanotte M.M., Lopiano L., Carpinella I. (2017). The Dominant-Subthalamic Nucleus Phenomenon in Bilateral Deep Brain Stimulation for Parkinson’s Disease: Evidence from a Gait Analysis Study. Front. Neurol..

[75] Johnsen E.L., Mogensen P.H., Sunde N.A., Østergaard K. (2009). Improved Asymmetry of Gait in Parkinson’s Disease with DBS: Gait and Postural Instability in Parkinson’s Disease Treated with Bilateral Deep Brain Stimulation in the Subthalamic Nucleus. Mov. Disord..

[76] Davis R.B., Õunpuu S., Tyburski D., Gage J.R. (1991). A Gait Analysis Data Collection and Reduction Technique. Hum. Mov. Sci..

[77] Evans E., Cook D. (2007). Case Study Evaluation of Body Weight-Supported Treadmill Training for Parkinsonian Gait. Int. J. Ther. Rehabil..

